# Preserved and Accessible: Quantifying PFAS in Formalin-Fixed and Paraffin-Embedded Tissues for Retrospective Exposure and Dose Assessment

**DOI:** 10.1021/acs.est.5c18596

**Published:** 2026-05-27

**Authors:** Kushal Biswas, Jennifer J. Schlezinger, Anila Bello, Dhimiter Bello

**Affiliations:** † Department of Biomedical and Nutritional Sciences, Zuckerberg College of Health Sciences, 14710 University of Massachusetts Lowell, Lowell, Massachusetts 01854, United States; ‡ Department of Environmental Health, School of Public Health, Boston University, Boston, Massachusetts 02118, United States; § Department of Public Health, Zuckerberg College of Health Sciences, 14710 University of Massachusetts Lowell, Lowell, Massachusetts 01854, United States

**Keywords:** PFAS, biomonitoring, LC−MS/MS, formalin-fixed tissues (FFT), FFPE, tissue recovery, retrospective exposure assessment

## Abstract

*Background:* Formalin-fixed (FFT) and formalin-fixed paraffin-embedded (FFPET) tissues represent an immense and underutilized resource for reconstructing historical chemical exposures. For persistent contaminants such as per- and polyfluoroalkyl substances (PFAS), these archived specimens could enable retrospective biomonitoring and tissue-level dosimetry, linking exposure history to disease outcomes. However, fixation and embedding may alter analyte stability and recovery, creating uncertainty in quantification. *Methods:* We have developed and tested a liquid chromatography–electrospray tandem mass spectrometry (LC-ESI-MS/MS) method to quantify six commonly found environmentally relevant PFAS (PFHxS, PFOA, PFOS, PFNA, PFDA, PFUnA) in mouse liver, kidney, ileum, and brain. Tissues from PFAS-exposed mice were divided into matched sets of flash-frozen tissues (FT), formalin-fixed (FFT), and formalin-fixed paraffin-embedded (FFPET) samples. PFAS recovery and loss were tracked through fixation, storage, and deparaffinization steps. *Results:* Across matched flash-frozen tissues, the mean sum of PFAS ranged from 279.2 ng/g in the brain to 22,628.4 ng/g in the liver. All PFAS were quantifiable in each matrix. Formalin fixation largely preserved PFAS, with recoveries of 76.7–83.8% relative to fresh tissue, while 12.6–19.9% of PFAS were detected in the formalin solution. Paraffin embedding resulted in further losses, yielding 57.4–75.1% recovery, with 6.1–15.9% loss during xylene and ethanol processing. Longer-chain PFAS (PFNA–PFUnA) exhibited greater retention than shorter-chain species (PFHxS), and tissues with higher PFAS burdens, such as liver, showed better recovery than low-burden tissues like brain and ileum. *Conclusion:* This work demonstrates the feasibility of quantifying PFAS in archived tissues and provides the first systematic insight into compound- and matrix-specific recovery patterns. Further validation is needed for short and ultrashort PFAS. Human tissues with much lower PFAS loads may experience proportionally greater analytical losses, requiring higher analytical sensitivity and application of correction factors for accurate dose estimation.

## Graphical abstract

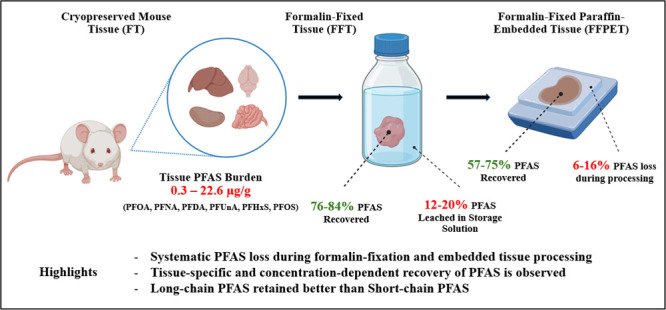

## 1 Introduction

Per- and polyfluoroalkyl substances (PFAS) are a broad class of synthetic organofluorine compounds defined by carbon chains fully or partially substituted with fluorine atoms.^1^ The exceptional stability of the carbon–fluorine (C–F) bond confers unique physicochemical properties on PFAS, including remarkable thermal and chemical stability, amphiphilicity, and notable oil and water repellency. This same stability also makes PFAS highly persistent in the environment and in biological systems.^2^ Over several decades, the landscape of PFAS has evolved significantly, initially dominated by long-chain perfluoroalkyl acids (e.g., C8 compounds like perfluorooctanoic acid (PFOA) and perfluorooctanesulfonic acid (PFOS)), which gained widespread use in products ranging from nonstick coatings and firefighting foams to textiles and food packaging materials.^3,4^ Subsequent legal and regulatory actions, combined with voluntary industry replacements, and increasing awareness of their environmental persistence and toxicity led to a shift toward shorter-chain PFAS and novel fluorinated alternatives; however, these replacement compounds often possess similar intrinsic properties, contributing to their continued global presence.^5,6^

Widespread human exposures to PFAS have continued for decades, and their biopersistence, bioaccumulation, and biomagnification in ecological systems, combined with their highly toxic profile, have raised significant concern for human health.^7,8^ PFAS exposure among humans was largely unnoticed for many decades, and nearly everyone has PFAS in their blood, currently typically within the 1–10 ng/mL serum range, although compound-specific distributions vary widely across populations and exposure settings.^9,10^ Recent U.S. biomonitoring data reported by ATSDR based on NHANES 2017–2018 show geometric mean blood concentrations of approximately 4.25 ng/mL for PFOS, 1.42 ng/mL for PFOA, 1.08 ng/mL for PFHxS, and 0.411 ng/mL for PFNA.^9^ In contrast, tissues provide information on organ-specific accumulation and dosimetry, which may diverge substantially from serum because PFAS partitioning is tissue- and compound-dependent.^11^ Individual PFAS levels and their profiles in human blood (and tissues) today are quite different from those of decades ago^12,13^ PFAS have been linked to an increased risk of testicular and kidney cancers, ulcerative colitis, impaired immune responses to vaccination, low birth weight, dyslipidemia, hypertensive disorders of pregnancy, thyroid disruption, hepatotoxicity, and nephrotoxicity, among other effects.^14–16^ It is reasonable to assume that most PFAS-related diseases of today reflect exposures of decades ago, which are no longer captured by current blood biomonitoring studies.

Biological sources, such as blood (serum or plasma), urine, breast milk, hair, and tissue samples (e.g., liver, kidney, brain, and intestine), can be used for PFAS biomonitoring and tissue dosimetry.^17^ Biofluid-based analysis provides easier access for biomonitoring as well as insights into the current body burden, and ongoing exposures. In contrast, tissue-based analysis offers significant additional advantages, including the ability to evaluate PFAS distribution and accumulation at the organ level, facilitate accurate dose–response analysis and target organ toxicity, and support the development and validation of physiologically based pharmacokinetic (PBPK) modeling.^11,18,19^ Freshly harvested and cryopreserved human tissues from surgical procedures are ideal for PFAS analyses as they represent the closest-to-normal physiological status of the tissue. However, analysis of freshly collected or cryopreserved tissues is uncommon and challenging to secure due to its demanding and complex logistics. Proper collection, storage, and transport of these tissue samples requires continuous access to low-temperature storage (e.g., −80 °C freezers or dry ice), which may be unavailable in field settings or resource-limited regions.^20,21^ Additionally, there is always a risk of analyte degradation during transportation.^22^

Formalin-fixed tissues (FFT) and formalin-fixed paraffin-embedded tissues (FFPET) are the standard methods for preserving biological tissue samples from decomposition and for archiving.^23^ These preparation methods for histopathological examination have ensured the preservation of millions of tissue samples over decades, utilized for histological analysis, pathological diagnosis, molecular analysis, gene expression profiling, immunochemistry, tissue banking, and forensic analysis.^24,25^

Both FFT and FFPET samples represent potentially underutilized resources that may supplement fresh or cryopreserved tissues for retrospective PFAS analysis, particularly when archival specimens provide the only available window into historical exposure trends over multiple decades. When interpreted with appropriate consideration for procedural losses, quantifying PFAS in FFPET could support exploratory investigations into the relationships between tissue-specific PFAS burdens and longitudinal health outcomes, potentially informing risk assessments or identifying high-exposure cohorts.^26^ This analytical approach may offer a preliminary framework for PFAS tissue dosimetry, which could eventually complement existing pathology and biochemistry data in biobanks to better understand molecular pathways potentially influenced by PFAS.^27^ Furthermore, while acknowledging the complexities of tissue processing, archived samples may provide a unique, albeit challenging, opportunity to study the long-term presence of PFAS species and their potential biochemical precursors within specific tissue microenvironments.^28,29^ Ultimately, comparative analysis across different tissues from the same individual may yield insights into PFAS pharmacokinetic profiles and their variation across different life stages and health statuses, helping to address data gaps stemming from the inherent scarcity of human tissue measurements.^30^

Although FFT/FFPET can provide valuable data on PFAS toxicology and pharmacology research, to our knowledge, no prior study has systematically quantified PFAS recovery from matched flash-frozen, formalin-fixed, and paraffin-embedded tissues. While most PFAS are chemically stable, the extensive processing steps that a tissue undergoes during its fixation and preservation raise the question of analyte loss during the multiple sample preparation steps or reduced accessibility to trapped PFAS inside the tissue. Comparison of unprocessed tissues with known PFAS to the processed tissues, robust controls, and adequate tissue mass are required for this purpose. The goal of this study is to develop and validate methods for analyzing PFAS in FFT/FFPET, representing a timely advancement in the analytical chemistry of PFAS. For this purpose, we used tissues from mice exposed to a known mixture of commonly found environmentally relevant PFAS.

## 2 Materials and Methods

### 2.1 Chemicals and Materials

We obtained high-purity deionized water (18MΩ) from a Milli-Q Direct 8 unit (Millipore Sigma). LC-MS grade solvents, including Methanol (Cat# BDH85800.400) and Xylene (histology grade, Cat # MSPP-RS4050), were purchased from VWR International (Radnor, PA). We also purchased LC-MS grade Ammonium Acetate, Ammonium Hydroxide, and Sodium Hydroxide from Sigma-Aldrich (Chicago, IL). The HPLC analytical column (Luna Omega PS C18, 3 μm, 100 Å, 100 × 4.6 mm, Part# 00D-4758-E0) and guard column cartridge (SecurityGuard PS C18, 4 × 3.0 mm, Part No. AJ0-7606) came from Phenomenex (Torrance, CA). A PFAS delay column (Ascentis Express 160 Å PFAS Delay, 2.7 μm, 50 × 4.6 mm, Cat# 53573-U) was purchased from Supelco, Millipore Sigma (Atlanta, GA). We obtained solid-phase extraction cartridges, Strata X-AW (33 μm polymeric weak anion; 60 mg/3 mL, Cat# 8B-S038-UBL) from Phenomenex Inc. (Torrance, CA) and Supelclean (ENVI-Carb SPE Tube, part#57092) from Supelco Inc. (Bellefonte, PA). Polypropylene LC-MS vials (SureSTART Polypropylene Microvials, Cat#6ESV9–1PP) were obtained from Fisher Scientific Company (Hanover Park, IL). All PFAS standards (linear isomers) and mass-labeled PFAS IS solutions (MPFAC-HIF-ES and MPFAC-HIF-IS) were purchased from Wellington Laboratories LLC (Wilmington, DE).

### 2.2 In Vivo PFAS Exposure to Animals

All animal studies were approved by Boston University’s Institutional Animal Care and Use Committee and were conducted in an American Association for the Accreditation of Laboratory Animal Care-accredited facility (Animal Welfare Assurance Number: A3316-01). Male and female PPARα knockout (null) mice expressing a human PPARα transgene (hPPARα) were used in these studies (generously provided by Dr. Frank Gonzalez, NCI).^52^ Experimental mice were generated from hPPARα heterozygous breeding pairs and genotyped to ensure expression of hPPARα. Mice (30–38 days of age) were acclimated for 1 week before exposure on a custom control diet based on the What We Eat in America (WWEIA; NHANES 2013–2014) analysis of adult dietary intake (Research Diets, New Brunswick, NJ; USDA, 2018). All analyses were performed exclusively on samples collected from mice maintained on this control WWEIA diet. During the exposure, they were then provided with one of two custom diets and given six PFAS (PFOA 41 ng/mL, PFNA 186 ng/mL, PFDA 51 ng/mL, PFUnA 56 ng/mL, PFHxS 40 ng/mL, PFOS 50 ng/mL) in their drinking water. Sucrose (0.5%) was added to the drinking water to encourage PFAS consumption, but the concentration was significantly lower than that in sugar-sweetened drinks (10–12%). Concentrated stock solutions of each PFAS were prepared in NERL water and then diluted in NERL water containing 0.5% sucrose. Great care was taken to minimize contamination of the vehicle drinking water. Glass water bottles with rubber stoppers (WTRBTL, Braintree Scientific, Inc., Braintree, MA) were labeled for PFAS drinking water and kept separate throughout the experiment. Water bottles and stoppers were washed using alkaline soap and scrub brushes that were also kept separate. After 6 weeks of exposure, PFAS-exposed mice were euthanized. Before euthanasia, the drinking water was switched to unamended water, and the mice were fasted for 6–8 h. At euthanasia, blood was collected via cardiac puncture, and serum was separated.

The liver, kidney, ileum, and brain were selected for PFAS analysis. All tissues were flash-frozen in liquid nitrogen. After collecting these tissues, they were weighed and aliquoted. Three different tissue aliquots from the same organ were set aside for three separate experiments. One aliquot of the tissue was stored at −80 °C and referred to as flash frozen tissue (FT). This aliquot was then analyzed directly for PFAS using the method described in the upcoming sections (Method A). Another aliquot of the cryopreserved tissue was formalin-fixed and referred to as formalin-fixed tissue (FFT). This tissue was then analyzed for PFAS using a different method (Method B). The final aliquot of cryopreserved tissue underwent both formalin fixation and paraffin embedding, resulting in formalin-fixed, paraffin-embedded tissue (FFPET). This tissue was analyzed for PFAS using yet another method (Method C).

### 2.3 Formalin Fixation and Paraffin Embedding

Cryopreserved tissues were carefully thawed on ice, and excess surface moisture was gently blotted without compression. To ensure optimal fixative penetration, a uniform thickness of 3–5 mm (50–100 mg) was maintained. Then, a brief 1 min rinse in cold phosphate-buffered saline (PBS; pH 7.4) was used to remove debris and cryoprotectants. For fixation, tissues were then fully immersed in freshly prepared 10% neutral buffered formalin (NBF) at room temperature (20–25 °C), maintaining a minimum fixative-to-tissue volume ratio of 10:1 and incubated for 48 h with occasional gentle agitation to ensure even distribution. After fixation, tissues were transferred to storage solution (70% ethanol) for 12 weeks. These are the formalin-fixed tissues (FFT) that were used for PFAS analysis.

Some of the FFTs were used for paraffin embedding. After fixation, these FFTs underwent a controlled dehydration process using a graded ethanol series, which gradually removes water while preserving tissue structure. Next, tissues were cleared by immersing them in xylene for 1 h, twice, to remove ethanol and prepare the tissue for complete paraffin infiltration. The cleared tissues were then infiltrated with molten paraffin wax (56–58 °C). To embed, tissues were carefully positioned within embedding molds containing fresh molten paraffin, followed by solidification at room temperature and then at a cold plate or 4 °C to achieve optimal block hardness using a Paraffin Embedding Console (Sakura Tissue-Tek TEC 6), The finished, labeled FFPE tissue blocks were then stored at room temperature in a dry environment. These are the formalin-fixed paraffin-embedded tissues (FFPET) that were used for PFAS analysis.

### 2.4 Preparation of Tissue Samples for Analysis

In Method A, a portion of cryopreserved unprocessed tissue (50–100 mg) underwent targeted PFAS analysis following a lab-established protocol. After measuring the tissue weight, the tissue was homogenized using a Fisherbrand Bead Mill 4 mini homogenizer (Cat#15-340-164), and a known amount of PFAS internal standard mix was added (SI Table S4). The tissue was then digested with a 10 mM sodium hydroxide solution in methanol for over 16 h with continuous shaking. Following digestion, the resulting mixture underwent centrifugation (12000 rpm/min for 20 min), and the supernatant was separated. The supernatant was then cleaned using solid-phase extraction steps to remove interfering lipids for further purification. Finally, the eluate, containing PFAS (2 × 1 mL of 2% ammonium hydroxide in methanol), was evaporated to near-dryness using a gentle stream of nitrogen, and then reconstituted with 200 μL of methanol. The eluent was then analyzed using a custom LC-ESI-MS/MS method to identify and quantify target PFAS.

In Method B, tissue samples were fixed in 10% neutral buffered formalin (24 h of rocking at room temperature, followed by storage at 4 °C until processing). To initiate the experiment, the storage solution (70% ethanol) was removed from the FFT, and the solution used to store the FFTs was also analyzed for PFAS to determine the total amount of PFAS loss during storage. To analyze this solution, an aliquot was collected, spiked with the internal standards mix, concentrated, and processed using the same analytical workflow as for tissue extracts prior to LC-ESI-MS/MS analysis. The FFTs were kept in PBS solution and then deionized water, with gentle agitation to facilitate the removal of excess storage solution from the surface. The tissue was then blot-dried and air-dried to eliminate excess water. Finally, the tissue was weighed and subjected to PFAS quantitation as described in Method A.

In Method C, FFPET blocks were taken and deparaffinized with xylene, which was collected to assess any PFAS loss during the process. The sample then underwent a series of ethanol–water washes to remove xylene and other residuals. Solvents from each washing step were preserved, spiked with internal standard mix, concentrated and analyzed later to identify any PFAS losses during the washing steps. After this step, the tissue was air-dried to remove excess water. Finally, the sample’s weight was measured, and PFAS quantification was performed according to Method A.

### 2.5 PFAS Chemical Analyses

The analytical method targeted the six PFAS administered to the mice: PFHxS, PFOA, PFOS, PFNA, PFDA, and PFUnA. All calibration standards and reported quantitative values correspond to the linear isomers of these target PFAS. No branched isomers were detected in the standards and the tissues of animals. SI Table S1 provides details on the analytes and other parameters for analysis, and SI Table S4 contains information on corresponding mass-labeled internal standards. It should be noted that perfectly matched mass-labeled internal standards were unavailable for PFOS and PFHxS; therefore, the closest available structurally similar isotope-labeled surrogates were utilized for their quantification, which may introduce minor biases in absolute matrix effect. Liquid chromatography–negative electrospray ionization-tandem mass spectrometry (LC-ESI-MS/MS) was used with an Applied Biosystems triple quadrupole mass spectrometer (Sciex API 4000 LC-MS/MS) to quantify PFAS congeners, following the isotope dilution method as previously reported.^31^ In brief, chromatographic separation occurred on a Shimadzu LC20 series stack with a Luna Omega PS C18, 100 × 4.6 mm analytical column from Phenomenex. The mobile phases consisted of 10 mM ammonium acetate in deionized water (A) and 10 mM ammonium acetate in methanol (B). The chromatographic gradient began at 10% B for the first minute, then increased to 65% B at 2 min, 99% B at 15 min, and remained at 99% B until 20 min, followed by 5 min of postcolumn equilibration. A 10 μL sample injection volume was used. To eliminate background PFAS contamination, we employed an online delay column (Ascentis Express 160 Å PFAS Delay, 2.7 μm, 50 × 4.6 mm). A diverter valve from VICI (Valco Instrument Co., Inc.) directed the front (0–3 min) and back ends (19–22 min) of the chromatographic run to waste. For data collection on the target set of PFAS, we used the scheduled MRM mode (SI Table S1).

### 2.6 Quality Control

To ensure analytical quality control, multiple measures were implemented throughout sample preparation and instrumental analysis. After every ten samples, a laboratory blank was included to monitor background contamination from solvents, reagents, and consumables. To evaluate potential matrix-derived interferences, unexposed control (blank) mouse tissues of liver, kidney, brain, and ileum were processed and analyzed alongside experimental samples. In addition, analytical accuracy was further verified using the NIST Aqueous Film-Forming Foam (AFFF) formulation reference material 8690, which contains representative long- and shorter-chain PFAS compounds. Standards were spread randomly across the sequence.^32^ The detection limits varied from 5 to 13 pg/mL in solution and 0.1–0.25 ng/g in tissues (SI Table S1). The limit of detection (LOD) and limit of quantification (LOQ) for each target analyte were determined based on a signal-to-noise (S/N) ratio of 3:1 and 10:1, respectively.

Given the diverse biochemical composition of the analyzed organs, varying degrees of matrix effects were expected during LC-ESI-MS/MS analysis. These effects were addressed using isotope-dilution quantification with mass-labeled internal standards added prior to extraction and cleanup. Quantification was performed using analyte-to-internal-standard peak area ratios, which corrected for extraction variability and helped normalize ion suppression or enhancement during electrospray ionization. Tissue-specific, matrix-matched calibration curves were prepared for liver, kidney, ileum, and brain matrices to further account for organ-dependent matrix effects. The internal standard recoveries reported in SI Table S4 represent apparent procedural recoveries after extraction and cleanup and should not be interpreted as independent measurements of matrix effects. Overall, internal standard recoveries ranged from 75.3 to 92.5%, and all calibration curves showed excellent linearity, with coefficients of determination above 0.999 (*R* ^2^ > 0.999). We analyzed 20% of the samples as true blind replicates. Reproducibility and injection-to-injection variability of PFAS quantification were also assessed (SI Table S2). An aqueous film-forming foam (AFFF) NIST Reference Material 8690 was used to compare against standards and measure analytical method biases (SI Table S3). The measured concentrations for individual PFAS were within −4.9 to +8.5% of the certified NIST reference material 8690 values (SI, Table S3), confirming the validity of the calibration standards and analytical workflow. We used tested and certified PFAS-free labware throughout the entire sample acquisition, processing, and analysis process. None of the target PFAS were detected above the method LODs in laboratory blanks or blank control tissues. Injection repeatability was evaluated using seven replicate injections of the same prepared representative FT extract within a single analytical batch. Injection-level precision was high across all tissues, with relative standard deviations (RSDs) ranging from 0.5% (liver) to 2.7% (brain) for total PFAS quantification (SI Table S2).

### 2.7 Statistical Analysis

All statistical analyses were performed in GraphPad Prism (version 10.5.0). Concentrations of individual species and the sum of six target PFAS (ΣPFAS) were normally distributed; therefore, all results are presented as the arithmetic mean (standard deviation, SD) for each tissue group (*n* = 10 samples per group). Normality was assessed using the Shapiro-Wilk test prior to ANOVA. With *n* = 10 per tissue group, the study had adequate to excellent power to detect large differences in recovery, particularly for comparisons involving liver and brain and for most FFPET contrasts (power, >0.8 – ∼1.0). However, power was limited for smaller intertissue differences, especially kidney vs ileum and other moderate FFT contrasts (power, 0.3–0.6). Thus, nonsignificant pairwise differences should not be interpreted as evidence of equivalence, but rather as reflecting limited sensitivity for effects smaller than about 1.3 pooled SD units. Recovery (%) was calculated as the ratio of PFAS in processed tissues (FFT and FFPET) to the flash-frozen tissues (FT) and is reported as the Mean (SD). One-way analysis of variance was used to determine whether there were statistically significant differences in the recovery of ΣPFAS and individual compounds across tissue types. Furthermore, a one-way analysis of variance with Tukey’s multiple comparisons tests was used to account for differences between tissue groups. Linear regression was used to evaluate the relationship between PFAS concentrations in FT and the corresponding percentage recoveries in FFT, FFPET, and processing media (storage solution, xylene, and ethanol–water). Simple linear regression analysis was performed for each tissue type and PFAS species.

## 3 Results

### 3.1 Measurement of Total PFAS in FT, FFT, FFPET, and Preparation Solutions

The total (sum of) and individual PFAS concentrations, quantified with all three methods, across different tissue conditions (FT, FFT, and FFPET) revealed varying recovery rates and distribution patterns. Table 1 and Figure 1 summarize the ΣPFAS concentration in the FT and the percentage recovered in FFT and FFPET, along with the PFAS lost during the formalin fixation process and FFPET processing. For fresh frozen tissues (FT), liver tissues exhibited the highest ΣPFAS concentration among unprocessed samples, measuring 22,628.4 ng/g, followed by kidney (2699.0 ng/g), ileum (1049.1 ng/g) and brain with the lowest concentration at 279.2 ng/g (Method A). Procedural losses and final recoveries for all tissues were determined using a mass-balance approach, with the measured FT concentration set as the 100% baseline.

**Table 1:** Sum of 6 Target PFAS (ΣPFAS) Concentrations and Recovery in Formalin-Fixed (FF) and Formalin-Fixed Paraffin-Embedded (FFPE) Tissues and ΣPFAS Losses during Preparation

tissue/tissue condition	mean (SD) ΣPFAS conc. in FT (ng/g)	mean (SD) ΣPFAS conc. in FFT (ng/g)	% ΣPFAS recovered in FFT mean (SD)	mean (SD) ΣPFAS conc. in FFPET (ng/g)	% ΣPFAS recovered in FFPET, mean (SD)	% ΣPFAS lost in storage solution, mean (SD)^a^	% ΣPFAS lost in FFPET processing mean (SD)^a^
liver (*n* = 10)	22628.4 (4946.5)	19113.6 (4472.2)	83.8 (3.2)	17002.0 (3310.6)	75.1 (2.1)	12.6 (2.2)	6.1 (2.2)
kidney (*n* = 10)	2699.0 (638.1)	2173.0 (559.9)	78.8 (4.2)	1925.5 (450.5)	68.2 (5.5)	16.3 (3.4)	7.7 (1.4)
ileum (*n* = 10)	1049.1 (239.9)	845.2 (226.0)	79.4 (4.4)	709.8 (174.3)	64.3 (6.2)	19.9 (3.7)	12.5 (3.2)
brain (*n* = 10)	279.2 (51.6)	216.8 (41.3)	76.6 (3.7)	163.4 (29.9)	57.4 (4.0)	17.3 (2.5)	15.9 (1.6)

a Mass-balance calculation: $\%{\mathrm{Loss}}_{\mathrm{Solvent}}=\frac{{\mathrm{Mass}}_{\mathrm{SolventExtract}}}{{\mathrm{Mass}}_{\mathrm{FT}}}\times 100$

**Figure 1:**
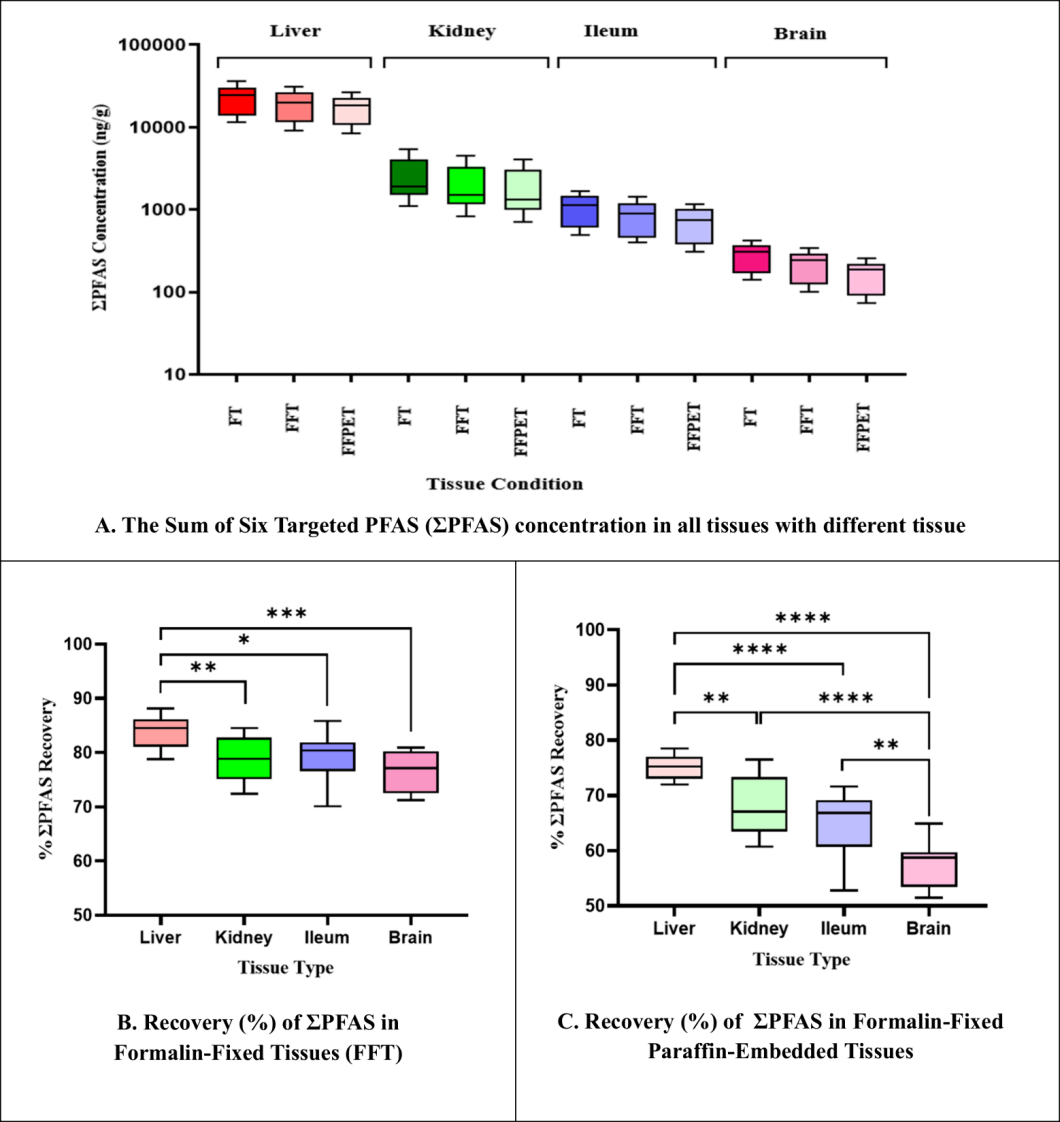
Percent (%) recovery of total PFAS in different types of formalin-fixed tissues (FFT) and formalin-fixed paraffin-embedded tissues (FFPET) compared with total PFAS measures in flash frozen tissues (FT) (Table 1). The six targeted PFAS are PFHxS, PFOA, PFOS, PFNA, PFDA, and PFUnA. Data points represent biological replicates, and horizontal lines indicate the median. Statistical significance was determined using a one-way analysis of variance with Tukey’s multiple comparison (B, C). Only significant *P*-values are shown above the plots as follows: *P < 0.05, **P < 0.01, ***P < 0.001, and ****P < 0.0001.

Formalin-fixed tissues (FFT) showed lower PFAS concentrations compared to fresh tissue, reflecting systematic loss during storage. Recovery of ΣPFAS in FFT, analyzed using Method B, was highest in liver (83.8%), followed by ileum (79.4%) and kidney (78.8%), with brain exhibiting the lowest retention at 76.6%. Statistical comparisons indicated that recoveries from kidney, ileum, and brain did not differ significantly from one another, but each was significantly lower than liver (Figure 1B, *P* < 0.05–0.001). Analysis of the storage solutions used during the initial preservation process showed additional PFAS losses ranging from 12.6 to 19.9% across the four tissues, with the brain showing the greatest loss. These findings demonstrate that PFAS stability in FFT varies considerably by tissue type, with liver showing the strongest retention and brain the weakest, highlighting the need for tissue-specific correction factors when quantifying PFAS in archived samples.

Formalin-fixed paraffin-embedded tissues (FFPET), processed according to Method C, showed a significant decrease compared to FT. The percentage of PFAS recovered in FFPET ranged from 57.4% in the brain to 75.1% in the liver, indicating substantial analyte loss during paraffin embedding and subsequent deparaffinization. Statistical analysis confirms that liver and kidney differ significantly from the ileum and the brain (Figure 1C, *P* < 0.001). Furthermore, analysis of solvents from the xylene deparaffinization and subsequent ethanol–water washes (as described in Method C) quantified additional PFAS loss during the FFPET processing steps. The loss ranged from 6.1% in the liver to 15.9% in the brain, beyond what was found in the storage solution. This total loss was most significant in tissues with lower PFAS levels, particularly in the brain and ileum, indicating that tissues with low PFAS content are more susceptible to procedural losses during histological preparation. Figure 2 visualizes the association between the ΣPFAS concentration in FT and % recovery in FFT for liver, kidney, ileum, and brain. The regression values indicate a moderate positive association, with the strongest association in brain tissue (*R* ^2^ = 0.74, *p* < 0.05) and the weakest in liver tissue (*R* ^2^ = 0.44, *P* < 0.05).

**Figure 2:**
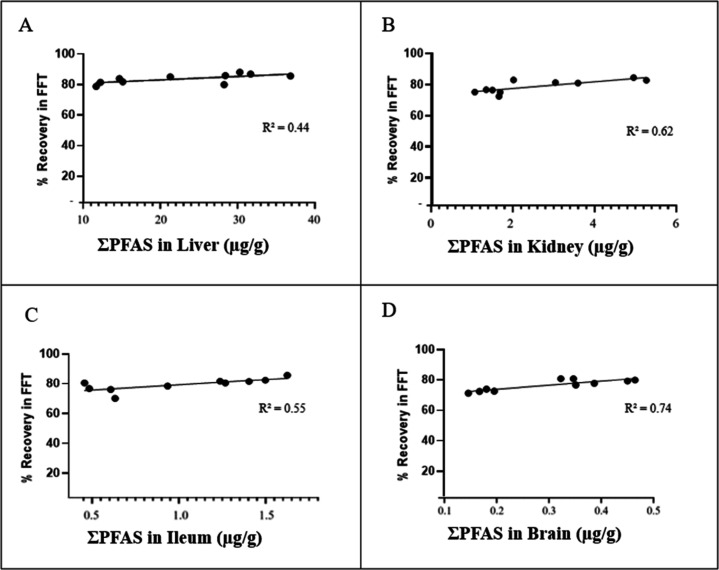
Linear regression analysis illustrating the relationship between ΣPFAS concentration measured in flash-frozen tissue (FT) and percentage recovery in formalin-fixed tissue (FFT) for (A) liver, (B) kidney, (C) ileum, and (D) brain. Each data point represents an individual tissue replicate. Solid lines indicate least-squares linear regression fits. The coefficient of determination (*R* ^2^) is reported for each tissue. (ΣPFAS includes PFHxS, PFOA, PFOS, PFNA, PFDA, PFUnA).

### 3.2 Measurement of Individual PFAS Concentrations in Different Tissues and Tissue Conditions

A detailed summary of the average concentrations of individual PFAS species (PFHxS, PFOA, PFOS, PFNA, PFDA, and PFUnA) in unprocessed tissues, along with their respective recovery in FFT and FFPET samples across liver, kidney, ileum, and brain tissues, is presented in Table 2. The initial concentrations of these PFAS species varied greatly depending on both the specific compound and the tissue type. Across all tissues, PFNA and PFOS showed the highest average concentrations, especially in liver tissue (3361.6 ng/g for PFOS and 12,826 ng/g for PFNA), indicating a tendency for these longer-chain PFAS to accumulate more; however, part of this observation can be explained by the much higher PFNA concentration in the mixture (Figure 3).

**Table 2:** Concentrations of Individual PFAS Species and Specific Recovery in Formalin-Fixed (FFT) and Formalin-Fixed Paraffin-Embedded Tissues (FFPET) Relative to Flash Frozen Tissues (FT) (*n* = 10/Group)

tissue type	liver (*n* = 10)			kidney (*n* = 10)			ileum (*n* = 10)			brain (*n* = 10)		
PFAS species	mean (SD) FT PFAS conc. (ng/g)	% PFAS recovered in FFT, mean (SD)	% PFAS recovered in FFPET, mean (SD)	mean (SD) FT PFAS conc. (ng/g)	% PFAS recovered in FFT mean (SD)	% PFAS recovered in FFPET, mean (SD)	mean (SD) FT PFAS conc. (ng/g)	% PFAS recovered in FFT mean (SD)	% PFAS recovered in FFPET mean (SD)	mean (SD) FT PFAS conc. (ng/g)	% PFAS recovered in FFT mean (SD)	% PFAS recovered in FFPET mean (SD)
PFHxS	583.8 (112.1)	80.5 (6.6)	60.6 (6.9)	426.6 (38.4)	77.4 (5.8)	63.5 (6.3)	31.2 (3.1)	73.7 (7.3)	46.8 (5.4)	19.2 (4.0)	72.4 (4.1)	49.8 (5.8)
PFOA	687.5 (101.2)	78.2 (4.9)	62.3 (8.3)	165.6 (38.2)	76.8 (6.1)	61.0 (6.8)	151.2 (25.1)	77.3 (4.3)	59.7 (5.5)	18.7 (5.4)	72.7 (4.6)	48.6 (5.2)
PFOS	3361.6 (614.2)	85.0 (4.7)	72.2 (8.4)	415.9 (66.8)	82.9 (7.1)	71.9 (7.6)	75.3 (7.7)	72.2 (5.0)	57.8 (2.5)	52.3 (12.2)	80.1 (5.5)	60.8 (5.1)
PFNA	12826.2 (3781.4)	83.8 (4.1)	77.1 (5.4)	1265.8 (237.7)	78.4 (5.4)	71.3 (6.2)	553.4 (211.3)	82.6 (4.7)	70.8 (5.2)	94.6 (25.0)	77.6 (4.6)	58.0 (6.6)
PFDA	2772.8 (651.0)	86.1 (3.2)	79.4 (4.1)	254.0 (27.1)	77.7 (4.8)	66.9 (4.3)	175.8 (23.4)	75.2 (5.0)	59.7 (5.5)	45.7 (21.8)	74.2 (3.6)	54.8 (2.2)
PFUnA	2395.8 (339.2)	81.5 (5.7)	72.2 (4.2)	171.1 (45.7)	76.9 (5.5)	61.6 (7.4)	62.1 (19.5)	71.0 (7.2)	57.7 (4.8)	48.7 (17.4)	75.6 (5.5)	60.9 (6.7)

**Figure 3:**
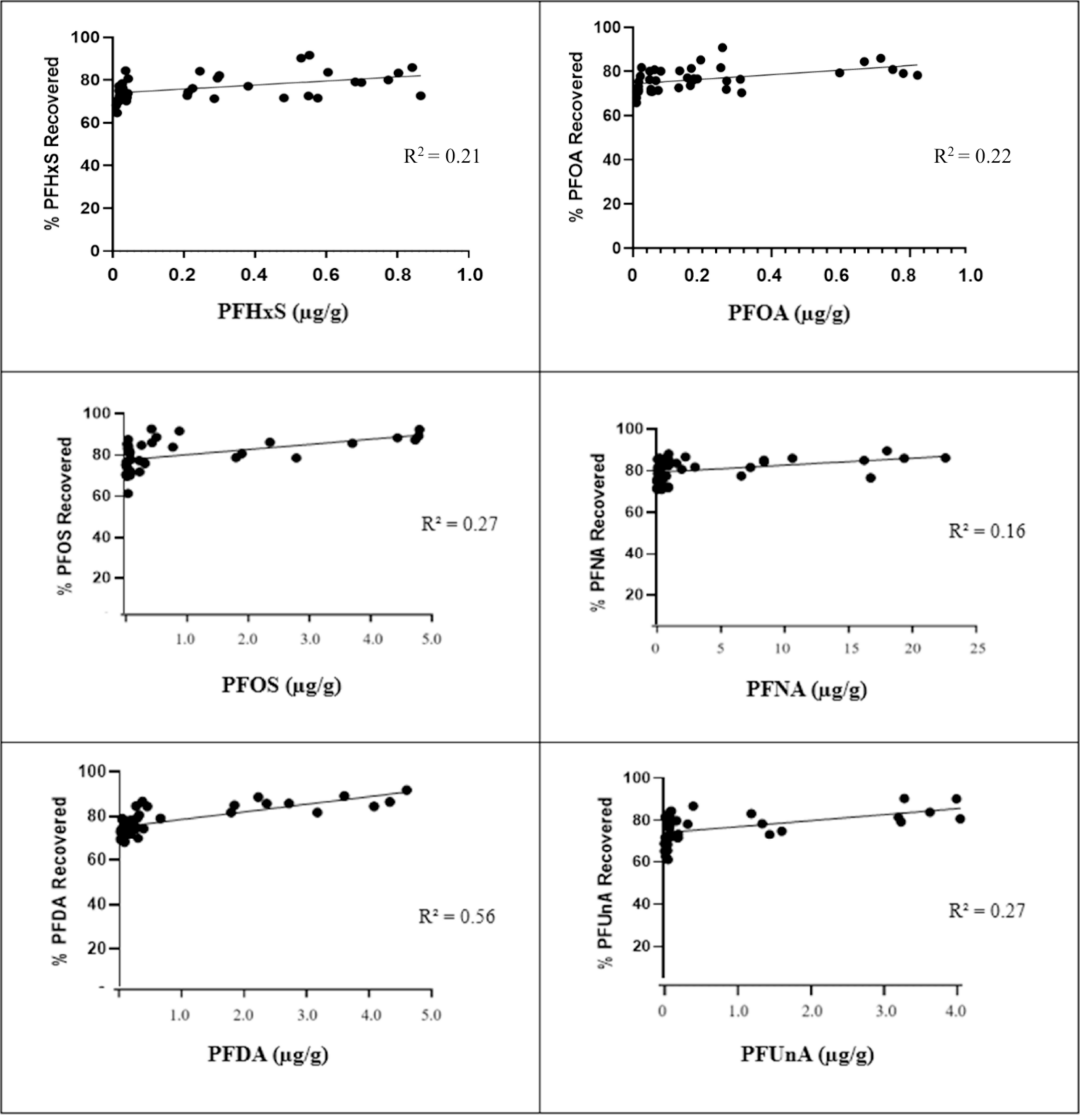
Linear regression analysis between individual PFAS concentration (*x*-axis) measured with % recovery (*y*-axis) across all formalin-fixed tissues (FFT). Each data point represents an individual tissue replicate. Solid lines indicate least-squares linear regression fits. The coefficient of determination (*R* ^2^) is reported for individual PFAS.

Among all tissues examined, the liver exhibited the highest PFAS concentrations in fresh tissue and consistently showed the greatest recovery after preservation. In formalin-fixed tissues (FFT), recovery of individual PFAS species ranged from 80.5% (PFHxS) to 86.1% (PFDA), with long-chain PFAS like PFOS, PFNA, PFDA, and PFUnA demonstrating significantly higher retention compared to shorter-chain PFAS (Figure 4). One-way ANOVA confirmed statistically significant differences among PFAS species in liver FFT samples (*P* = 0.007). After paraffin embedding (FFPET), liver PFAS recoveries decreased but remained relatively high, ranging from 60.6 (PFHxS) to 79.4% (PFDA) (Figure 5). The most notable reduction occurred with shorter-chain PFAS, whereas long-chain PFAS showed stronger retention, indicating greater stability during dehydration and deparaffinization.

**Figure 4:**
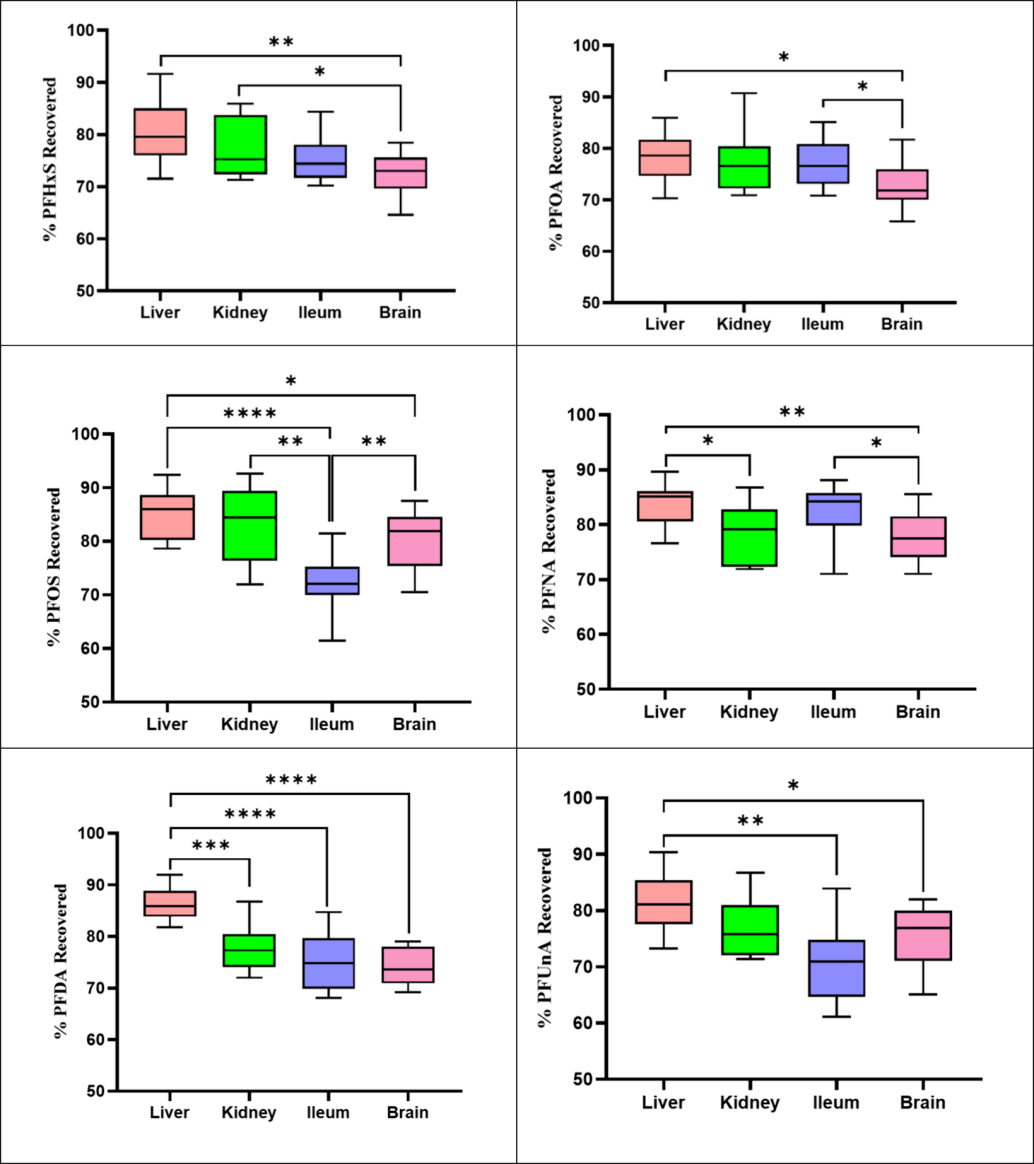
Recovery (%) of individual PFAS across different formalin-fixed tissues (FFT) samples (*n* = 10/group). Individual PFAS concentration is available in the supplementary table, and the (%) PFAS recovery (mean) is presented in Table 2. Each data point represents a biological replicate, and horizontal lines indicate the median values. Statistical significance was determined using a one-way analysis of variance with Tukey’s multiple comparison test for within-group comparisons. Only significant *P*-values are shown above the plots as follows: *P < 0.05, **P < 0.01, ***P < 0.001, and ****P < 0.0001.

**Figure 5:**
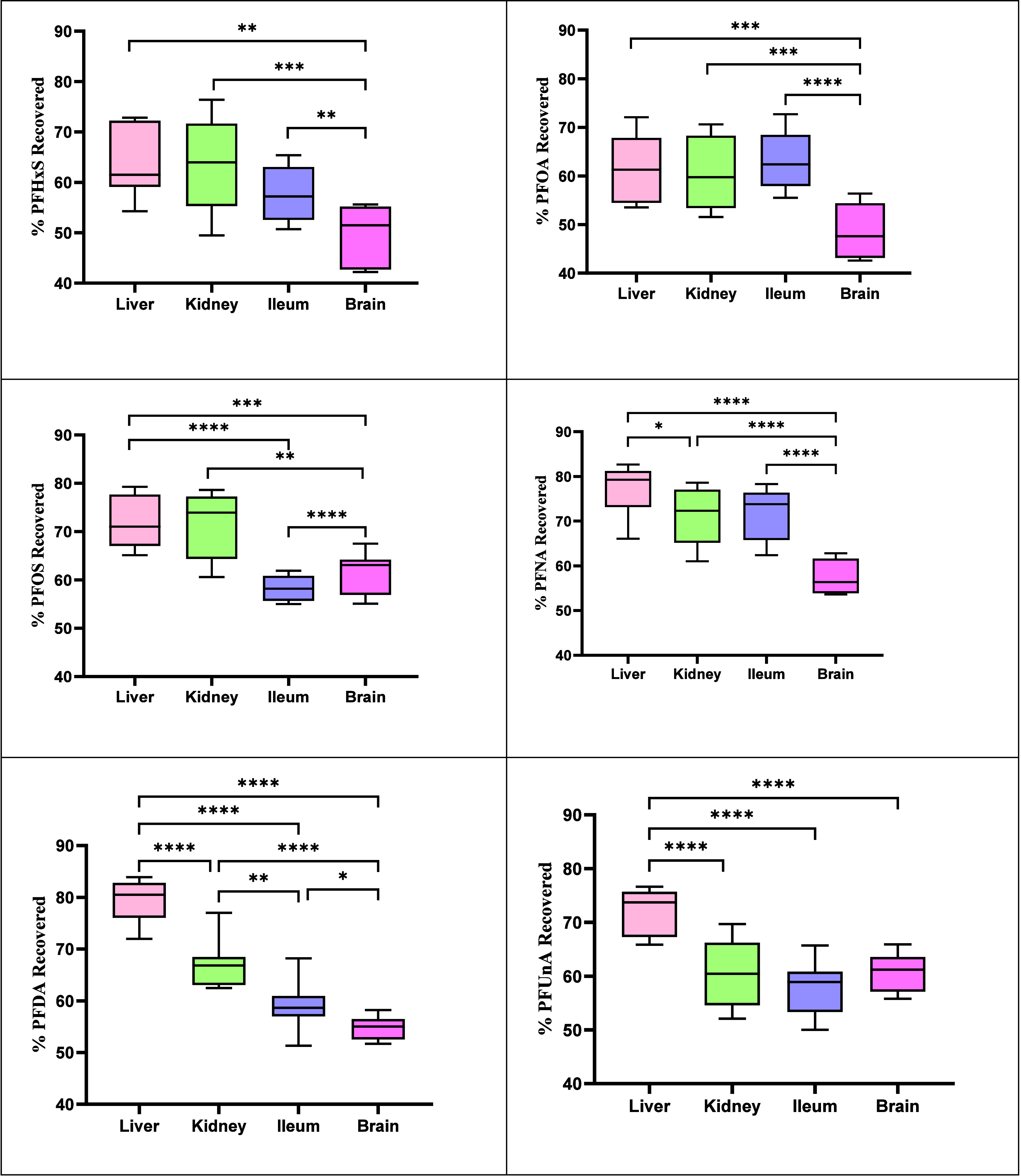
Recovery (%) of individual PFAS across different formalin-fixed paraffin embedded tissue (FFPET) (*n* = 10/group). Individual PFAS concentration is available in the Supplementary table, and the (%) PFAS recovery (mean) is presented in Table 2. Data points represent biological replicates, and horizontal lines indicate the median. Statistical significance was determined using a one-way analysis of variance with Tukey’s multiple comparison test for within-group comparisons. Only significant *P*-values are shown above the plots as follows: *P < 0.05, **P < 0.01, ***P < 0.001, and ****P < 0.0001.

In kidney tissue, FFT recoveries were moderately high and consistent across PFAS species, ranging from 76.8% (PFOA) to 82.9% (PFOS). Compared to the liver, the kidney showed slightly lower FFT recoveries and more variability among species. Supporting this, one-way ANOVA found no significant differences in recovery among PFAS species in kidney FFT samples (*P* = 0.182), indicating a more uniform effect of fixation across compounds (Figure 4). Paraffin embedding resulted in a greater decrease in recovery, with FFPET values ranging from 61.0% for PFOA to 71.9% for PFOS. PFHxS and PFOA consistently exhibited lower retention than long-chain compounds, suggesting they are more vulnerable to solvent-mediated losses during embedding and deparaffinization. Despite lower recovery rates, kidney tissue exhibited less species-dependent variation than other organs (Figure 5).

The ileum had lower initial PFAS concentrations compared to liver and kidney but still maintained relatively high recoveries after formalin fixation. FFT recoveries ranged from 71.0 (PFUnA) to 82.6% (PFNA), with statistically significant differences among PFAS species (*P* = 0.001; Figure 4), demonstrating effective preservation during fixation despite the lower PFAS levels. In contrast, paraffin embedding caused substantial additional losses, especially for shorter-chain PFAS, with PFHxS recovery dropping to 46.8% and PFOA to 59.7% (Figure 5). Long-chain PFAS maintained higher recoveries (up to 70.8% for PFNA), indicating compound-specific tissue retention. These results show that tissues with lower PFAS loads are more susceptible to solvent-mediated analyte loss during histological processing.

The brain tissue had the lowest initial PFAS levels among all the organs examined and was most affected by processing-related losses. In FFT samples, recoveries ranged from 72.4% (PFHxS) to 80.1% (PFOS), with significant species-dependent differences (*P* = 0.003; Figure 4), indicating good retention after fixation. However, FFPET processing caused the most substantial losses observed in this study, with recoveries dropping to 48.6–60.9%, especially for shorter-chain PFAS (PFHxS and PFOA; Figure 5). Shorter-chain PFAS experienced greater losses than long-chain PFAS. The notable decrease in recovery in brain tissue likely results from its lower PFAS burden and unique matrix composition, which together increase analyte partitioning into processing solvents during embedding and deparaffinization.

For most PFAS species analyzed, a general trend of a modest increase in recovery with higher tissue concentrations is discernible (Figure 3). A slightly positive but overall weak (*R* ^2^ = 0.16–0.27, liver, kidneys, ileum) or moderate (brain, *R* ^2^ = 0.56) association was observed between the concentration of individual PFAS species in unprocessed FT and their respective recoveries in FFT (Figure 3). The strength of the association varies among different PFAS species. Statistically significant differences in recovery were found among tissues for PFHxS, PFOS, PFNA, PFDA, and PFUnA. PFOA was the only species for which the recovery did not show a statistically significant difference among tissues. Brain and ileum trended consistently toward lower recoveries for most PFAS species.

## 4 Discussion

This study evaluates a critical analytical pathway for the retrospective quantification of PFAS in preserved biological matrices. This work represents a systematic quantification of PFAS recovery from both formalin-fixed tissues (FFT) and formalin-fixed paraffin-embedded tissues (FFPET). While PFAS analysis has to-date relied on fresh or cryopreserved specimens, our results demonstrate that archived pathology specimens, often accompanied by extensive clinical and demographic data, can serve as viable resources for tissue-specific dosimetry. By establishing quantitative boundaries for analyte recovery in processed tissues, this work enables new opportunities for mapping historical exposure trends and spatial distribution profiles across decades of archived samples. Leveraging these vast repositories provides a cost-effective means to explore the longitudinal relationship between tissue-level PFAS burdens and specific disease outcomes, addressing a significant data gap in contemporary environmental health research.

### 4.1 Impact of Tissue Processing on Total PFAS Concentrations

The results show that standard histological preservation methods inherently alter the apparent tissue burden of PFAS, underscoring the importance of accounting for processing effects when analyzing archived specimens. PFAS loss during formalin fixation is plausibly driven by diffusion into the aqueous storage medium, particularly as protein structures undergo denaturation and cross-linking.^33,34^ In fresh tissues, legacy PFAS are heavily sequestered by electrostatic and hydrophobic interactions with abundant tissue proteins, such as albumin and liver fatty acid-binding proteins (L-FABP), which exhibit strong association constants (*K*_a_ ranging from 10^4^ to 10^6^ M^–1^.^34–36^ Formaldehyde fixation relies on the covalent cross-linking of primary amine residues (e.g., lysine and arginine).^37^ Because these specific positively charged basic residues are critical for the electrostatic anchoring of the anionic PFAS carboxylate and sulfonate headgroups, their chemical neutralization during cross-linking likely reduces the overall PFAS binding affinity, facilitating diffusion-driven leaching into the aqueous storage solution. This behavior aligns with the amphiphilic nature of PFAS, which facilitates partitioning between biological tissues and bulk solutions.^1^ Factors such as fixation time, tissue surface-area-to-volume ratio, and agitation probably influence this process. Additional analyte loss during FFPE processing can be attributed to the sequential exposure of tissues to organic solvents during dehydration and clearing. Ethanol- and xylene-based steps disrupt residual aqueous compartments and promote PFAS partitioning into solvent phases, particularly for compounds that are not irreversibly sequestered within cross-linked protein networks.^1,38^ Aldehyde-induced protein cross-linking may further modify PFAS binding affinities, either transiently releasing compounds prior to fixation completion or immobilizing them within the matrix, depending on tissue composition and PFAS structure.^39–41^ Together, these mechanisms indicate that PFAS recovery from preserved tissues reflects a balance between diffusion-driven loss and matrix sequestration, rather than simple analytical inefficiency.

### 4.2 Tissue-Dependent Recovery of Total PFAS

Fundamental differences in tissue composition and microenvironment best explain variability in PFAS recovery across tissues. Lipid-rich tissues, such as the brain, provide a matrix in which PFAS are more susceptible to solvent-mediated redistribution during dehydration and clearing, as organic solvents preferentially extract analytes from hydrophobic compartments.^40,42^ . In contrast, the liver’s high protein and phospholipid content likely allows for stronger protein binding and sequestration of long-chain PFAS, leading to greater retention during histological processing.^43^ Notably, this observed recovery hierarchy (liver > kidney > brain) closely mirrors established in vivo PFAS tissue distribution patterns in both rodents and humans, demonstrating that the intrinsic tissue-binding capacities that govern physiological bioaccumulation also dictate analyte retention during chemical preservation.^11,44,45^ Although formalin cross-linking alters protein conformation, these interactions may still provide some protection against solvent-driven loss. Importantly, the lower recovery observed in the brain is a true matrix-driven partitioning effect rather than an analytical artifact of sample size. Because the brain aliquots (77–100 mg) were comparable to or larger than the other tissue types, absolute analyte masses remained well above the limit of detection. The kidney represents a distinct case, where PFAS recovery appears less sensitive to compound-specific physicochemical differences. Renal tissue expresses a broad complement of transporters, including organic anion transporters (e.g., OAT1, OAT2), that regulate PFAS handling through active uptake, secretion, and reabsorption.^46^ This dynamic equilibrium, combined with comparatively lower proteomic sequestration, may result in a more uniform PFAS distribution and recovery across species. This intermediate behavior also aligns with published studies showing kidney PFAS levels between liver and brain, supporting the link between biological partitioning and observed recovery trends.^18^ These findings highlight that tissue architecture, binding capacity, and biological function collectively govern PFAS fate during preservation, emphasizing the necessity of tissue-specific considerations when interpreting archived samples.

### 4.3 PFAS Species-Specific Recovery Patterns in Tissues

Beyond tissue-level effects, PFAS recovery is strongly influenced by compound-specific physicochemical properties. Chain length, functional group chemistry, and hydrophobicity/lipophilicity collectively determine the extent to which PFAS associate with proteins, membranes, or solvent phases during processing.^1,2^ Longer-chain PFAS exhibit stronger protein-binding affinities and more favorable hydrophobic interactions, enhancing their sequestration within cross-linked tissue matrices and reducing solvent-mediated loss (i.e., protein–water partitioning > membrane–water partitioning).^47^ Structural studies further demonstrate that fatty acid–binding proteins preferentially accommodate longer-chain PFAS through stable hydrophobic interactions, whereas shorter-chain PFAS bind more weakly and are therefore more vulnerable to redistribution.^48^ Shorter-chain PFAS, in contrast, exhibit greater aqueous solubility and reduced affinity for tissue proteins, rendering them particularly susceptible to loss during fixation and solvent exposure. The comparatively uniform behavior of PFOA across tissues may reflect its intermediate chain length and balanced partitioning behavior, resulting in less pronounced sensitivity to matrix composition. These observations reinforce the importance of compound-specific recovery assessment and caution against assuming uniform behavior across PFAS classes in preserved tissues.

### 4.4 Association between PFAS Tissue Concentration and Recovery

PFAS recovery from preserved tissues appeared to be concentration dependent, consistent with general principles of bioanalytical recovery. At higher tissue burdens, such as those observed in the liver, recovery rates were more stable, whereas lower-burden tissues showed proportionally greater losses during processing. This pattern may reflect a combination of matrix-specific retention, tissue composition, and concentration-dependent partitioning into formalin, ethanol, and xylene processing solutions. This concentration dependence has important implications for human biomonitoring, as PFAS levels in human tissues are typically substantially lower than those observed in controlled animal exposure studies.^49^ Our recovery data reveals that lower initial FT concentrations were associated with lower PFAS retention, particularly in brain and some ileum samples. At these lower tissue burdens (∼100–300 ng/g), process-related losses represented a larger fraction of the total analyte mass, contributing to contributing to lower percentage losses, often exceeding 40% in FFPET. In such contexts, processing-related losses may lead to false nondetects even when sensitive analytical platforms are employed, complicating exposure reconstruction and risk assessment. These findings emphasize the need for concentration-aware interpretation and, where possible, correction strategies when applying archived tissues to retrospective PFAS biomonitoring.

### 4.5 Study Limitations

The study focused on a specific set of well-known legacy PFAS compounds, which are of high concentration, have long half-lives, and exhibit higher tissue bioaccumulation rates than the newer generation of shorter-chain PFAS (C1–C4) that are found at increasingly higher frequency in humans in recent analyses.^11,49^ While the current data suggest that similar recoveries in FFT and FFPET may be observed with a broader range of PFAS species of comparable structure and length, the recovery data cannot be automatically extended to this wider range, especially shorter-chain PFAS and fluorotelomer alcohols. Experimental validation of other representative PFAS species (C1–C6) is needed, in part because the current data suggests more losses are likely for the shorter-chain PFAS. Our data defines recovery under controlled short-term fixation and storage conditions (12 weeks); they should not yet be extrapolated quantitatively to archival intervals of many years or decades without dedicated stability studies. We acknowledge that for truly archival human tissues, contamination introduced by historical preservation reagents, storage vessels, paraffin, or laboratory handling cannot be excluded retrospectively with the same certainty as in a controlled animal experiment. Furthermore, we tested PFAS recovery from only four major organs- liver, kidney, brain, and ileum. It is possible that the current observations may not extend to all organs, e.g., the lungs and bones/bone marrow, and additional experimental validation may be required in future studies. The tissue burdens achieved here exceed most reported human background organ concentrations (total PFAS ∼ 5.7–303.0 ng/g) and therefore should be interpreted primarily as a controlled recovery model rather than a direct surrogate for average human exposure.^11,49,50^ Along with that, this study quantified only six targeted terminal PFAS; we did not measure precursor compounds or transformation products. Consequently, it remains unclear whether precursor loss, transformation, or differential extraction occurred during the fixation and embedding processes.

## 5 Implications

This study provides evidence that PFAS can be reliably extracted and analyzed from archived formalin-fixed and formalin-fixed paraffin-embedded tissues, effectively transforming decades-old pathology collections into viable resources for retrospective biomonitoring. While histological processing inherently induces solvent-mediated analyte loss, our findings establish that FFPET analysis remains robustly reliable for tissues with higher initial PFAS burdens. At lower concentrations, accelerated percentage losses occur, necessitating careful data interpretation. To accurately reconstruct original tissue dosimetry, we recommend a correction strategy that uses tissue- and compound-specific regression-based adjustment factors, alongside parallel chemical analysis of storage solutions whenever available.

This approach is immediately applicable for reconstructing exposures in highly exposed occupational cohorts, point-source communities, and wildlife populations lacking historical serum samples. Future research must enhance mass spectrometric sensitivity for lower-background exposures, minimize procedural contamination, and expand the analytical suite to include short-chain and precursor PFAS. Ultimately, unlocking these archival “biological time capsules” allows epidemiologists to directly link historical tissue dosimetry to long-latency diseases, strengthening risk assessments, informing workers’ compensation, and supporting data-driven environmental health policies.

## Supplementary Material



## Notes

Additional details on the analytical methodology and quality control, including the target PFAS list, MRM transitions, MS parameters, detection and quantification limits, corresponding internal standards, instrumental repeatability data, analytical bias relative to NIST SRM 8690, and apparent procedural recoveries for the PFAS internal standards (PDF)
