# Supplementary material for: Preserved and Accessible: Quantifying PFAS in Formalin-Fixed and Paraffin-Embedded Tissues for Retrospective Exposure and Dose Assessment
Source: Environ Sci Technol. 2026 May 27;60(22):16122–34. doi: 10.1021/acs.est.5c18596 (PMC13262055; doi:10.1021/acs.est.5c18596)
Supplement: Supplementary file 1 [file es5c18596_si_001.pdf]

Title: Preserved and Accessible: Quantifying PFAS in Formalin-Fixed and Paraffin-Embedded Tissues for Retrospective Exposure and Dose Assessment

Authors

Kushal Biswas[1], Jennifer J. Schlezinger[2], Anila Bello[3], Dhimiter Bello[1*]

[1]Department of Biomedical and Nutritional Sciences, Zuckerberg College of Health Sciences, University of Massachusetts Lowell, Lowell, MA-01854, USA

[2]Department of Environmental Health, School of Public Health, Boston University, Boston, MA-02118, USA

[3]Department of Public Health, Zuckerberg College of Health Sciences, University of Massachusetts Lowell, Lowell, MA-01854, USA

***Corresponding Author:**

Dhimiter Bello, ScD, MSc

Email: Dhimiter_Bello@uml.edu

Table of Contents:

Page 2: Table S1: PFAS target list, respective MRMs, MS instrument parameters, detection limits, and corresponding internal standards

Page 3: Table S2: Reproducibility of PFAS quantification assessed by duplicate LC–MS/MS injections of a Flash-frozen Tissue (FT)

Page 4: Table S3: Analytical method biases relative to NIST SRM 8690 for analytes with available standards

Page 5: Table S4: (%) Recovery of the PFAS Internal Standards (IS) in the samples and their corresponding Analytes (PFAS)

Table S1: PFAS target list, respective MRMs, MS instrument parameters, detection limits, and corresponding internal standards

Nr	PFAS Names	Abbreviation / Common Name	Molecular Weight (g/mol)	MS/MS Parameters						Retention Time (min)	In-solution PFAS LOD (ng/ml)	In-solution PFAS LOQ (ng/ml)	Tissue PFAS LOD (ng/g)	Tissue PFAS LOQ (ng/g)
				Molecular Ion (Q1)	Product Ion (Q3)	Delustering Potential (Volts)	Entrance Potential (Volts)	Collision Energy (Volts)	Exit Potential (Volts)					
1	Perfluorooctanoic acid	PFOA	414.07	413.0	168.6	-40	-10	-24	-14	9.7	<0.006	<0.018	<0.11	<0.16
2	Perfluorohexanesulfonic acid	PFHxS	400.11	399.0	98.9	-80	-10	-70	-5	8.7	<0.007	<0.021	<0.13	<0.19
3	Perfluorononanoic acid	PFNA	464.07	463.0	419.0	-40	-10	-15	-10	11.9	<0.006	<0.018	<0.11	<0.16
4	Perfluorooctanesulfonic acid	PFOS	500.13	499.0	98.8	-80	-10	-60	-10	10.7	<0.013	<0.032	<0.25	<0.33
5	Perfluorodecanoic acid	PFDA	514.08	513.0	469.0	-45	-10	-15	-10	12.1	<0.005	<0.015	<0.10	<0.15
6	Perfluoroundecanoic acid	PFUnA	564.08	563.0	519.0	-40	-10	-15	-10	12.8	<0.005	<0.015	<0.10	<0.15

Table S2. Instrumental repeatability of the LC-ESI-MS/MS method, determined by seven replicate injections of a single tissue extract (n=7)*

Sample Injection	Liver FT (ng/g)	Kidney FT (ng/g)	Ileum FT (ng/g)	Brain FT (ng/g)
1st Injection	20 916.1	5 454.6	1 658.4	423.8
2nd Injection	20 675.6	5 526.2	1 685.3	401.5
3rd Injection	20 785.2	5 438.1	1 677.2	408.6
4th Injection	20 898.5	5 610.8	1 711.4	425.2
5th Injection	20 907.3	5 466.2	1 692.5	411.3
6th Injection	20 895.2	5 478.4	1 607.6	419.4
7th Injection	21 055.6	5 385.5	1 642.8	438.6
Mean	20 876.2	5 480.0	1 667.9	418.3
SD	109.6	66.3	32.2	11.4
% RSD	0.5	1.2	1.9	2.7

*Values represent intra-day injection repeatability based on seven replicate injections of the same prepared FT extract

Table S3: Analytical method biases relative to NIST SRM 8690 for analytes with available standards, based on three independent replicate analyses (n=3).

PFAS Standard	NIST Reference Materials 8690 (ng/ml ± SD)	Lab Calculated (n=3) (ng/ml ± SD)	Bias (%) = ((Lab Calculated - NIST Value) / NIST Value) × 100
PFBA	8.17 ± 1.27	8.86 ± 0.09	8.45
PFHxA	23.19 ± 2.05	22.67 ± 0.59	- 2.24
PFHpA	7.12 ± 2.91	7.55 ± 0.84	6.04
PFOA	24.92 ± 6.14	26.37 ± 1.88	5.82
PFPrS	23.74 ± 1.27	22.57 ± 0.97	- 4.93
PFBS	37.18 ± 2.53	37.82 ± 2.99	1.72
PFPeS	32.15 ± 6.69	31.25 ± 0.88	- 2.80
PFHxS	20.75 ± 1.94	22.13 ± 1.06	6.65
PFOS	15.25 ± 1.32	16.10 ± 1.50	5.57
6:2 FTS	17.45 ± 5.20	16.92 ± 1.71	-3.03
AVERAGE			2.12

Table S4: (%) Recovery of the PFAS Internal Standards (IS) in the samples and their corresponding Analytes (PFAS)

Serial	PFAS Names	Abbreviation / Common Name	Corresponding Internal Standard (Mass Labeled)	IS Mix Composition (ng/ml)	% IS Recovered
1	Perfluorooctanoic acid	PFOA	PFOA [M+2]	2.0	88.1
2	Perfluorohexanesulfonic acid	PFHxS	PFHxA [M+2]	2.0	75.3
3	Perfluorononanoic acid	PFNA	PFNA [M+5]	1.0	87.6
4	Perfluorooctanesulfonic acid	PFOS	PFOA [M+2]	1.0	88.1
5	Perfluorodecanoic acid	PFDA	PFDA [M+2]	1.0	92.5
6	Perfluoroundecanoic acid	PFUnA	PFUnA [M+2]	1.0	92.2
